# *Toxicodendron vernicifluum* Stokes extract inhibits solid tumor growth and lung metastasis of 4T1 murine mammary carcinoma cells in BALB/c mice

**DOI:** 10.1371/journal.pone.0241805

**Published:** 2020-11-05

**Authors:** Hyun Sook Lee, Jae In Jung, Kyeong-Hee Kim, Sang Jae Park, Eun Ji Kim

**Affiliations:** 1 Department of Food Science & Nutrition, Dongseo University, Busan, Korea; 2 Regional Strategic Industry Innovation Center, Hallym University, Chuncheon, Korea; 3 Medience Co. Ltd., Chuncheon, Korea; Chung Shan Medical University, TAIWAN

## Abstract

*Toxicodendron vernicifluum* Stokes has long been used as a food supplement and traditional herbal medicine in East Asia. We applied a new extraction method to produce *Toxicodendron vernicifluum* Stokes extract (TVSE), that doesn’t contain urushiol (an allergenic toxin) but dose have higher levels of some flavonoids such as fustin and fisetin. This study was conducted to investigate the anticancer effects of TVSE in an *in vivo* system. Fifty BALB/c mice were acclimated for one week and then injected with 4T1 murine mammary carcinoma cells in mammary fat pads. After 7 days, the mice were randomly divided into 5 groups, and orally administered with 0, 50, 100, 200 or 400 mg of TVSE/kg body weight (BW)/day for 20 days. TVSE reduced tumor volume and weight dose-dependently. The expression of Ki67 was significantly reduced and the number of TUNEL-positive apoptotic cells was significantly increased in the TVSE-treated group over 100 mg/kg BW/day. While tumor nodules were not found in the liver, but only in lungs, the number of tumor nodules was reduced in a dose-dependent manner in the TVSE treated groups compared to the control group. In breast tumors, expression of platelet endothelial cell adhesion molecule (PECAM-1) and vascular endothelial growth factor (VEGF) was reduced by TVSE treatment. TVSE treatment significantly suppressed mRNA expression in tumors of matrix metalloproteinase (MMP)-2, tissue inhibitor of metalloproteinase (TIMP)-1, urokinase-type plasminogen activator (uPA), intercellular adhesion molecule (ICAM)-1, and vascular cell adhesion molecule (VCAM)-1 while increasing plasminogen activator inhibitor (PAI)-1. These results suggest that TVSE is potentially beneficial for the suppression of breast cancer growth and its-associated lung metastasis.

## Introduction

Among women, breast cancer is the most commonly diagnosed cancer (24.2% of all cancer) and the leading cause of cancer death (15.0% of deaths) worldwide [1]. Breast cancer is a very heterogeneous disease and the etiology is still unclear. Early detection and early treatment can increase survival. However, metastasis to other tissues occurs in a large number of patients, especially in triple-negative (TN) breast cancer [2]. More than 90% of breast cancer deaths are due to metastasis-related complications [3]. Breast cancer cells can metastasize to diverse organs including lung, liver, bone, brain, etc., and once resident can proliferate into macroscopic masses, leading ultimately to death [4]. Five-year survival rates for distant metastasis breast cancer patients are less than 20% [5].

Metastasis is frequently a final and fatal step in the progression of solid tumors. The metastatic process consists of tumor cell intravasation, survival in circulation, extravasation into a distant organ, angiogenesis and uninhibited growth [6]. When cancer cells metastasize to other distant organs, microenvironmental factors work to overcome many barriers. In particular, when breast cancer cells metastasize from primary tumors to specific tissues, communication between the disseminated tumor cells and the resident stromal cells in those colonized tissues is varied but thought to be important to tumor progression. There are a variety of components that make up the microenvironment of tumors such as growth factors, immune cells, cytokines, chemokines, extracellular matrix (ECM), tumor-associated macrophages, and cancer-associated fibroblasts [7].

The mechanisms that determine which organs become metastasized by breast cancer are complex and influenced by many factors. One of them is the molecular subtype. Breast cancer is subdivided into four major clinical subtypes based on gene expression profiles and receptor status (estrogen receptor (ER), progesterone receptor (PR), human epidermal growth factor receptor 2 (HER2) and proliferative status as assessed by Ki67 [8]. These clinical subtypes can be classified as luminal A (ER +/PR +), luminal B (ER +/PR +/HER2-/+/Ki67 +), HER2 overexpression (ER-/PR-/HER +) and basal-like/TN (ER-/PR-/HER2-). Bone is the most common site of metastasis in all subtypes, but TN breast cancer is most likely to metastasize to the lung [9]. However, the timing and mechanism by which breast cancer molecular subtypes can affect metastasis to the lung are still unknown. Endeavors to understand the molecular mechanisms that induce breast cancer metastasis in the lung and to incorporate it into new therapies continue [10].

Recently, there is active interest in using products of natural medicinal plants that have anticancer effects and do not have cytotoxic values for cancer therapy or to overcome cancer cell drug resistance. Among these, taxol analogues, vinca alkaloids, and podophyllotoxin analogues play an important role in the treatment of some cancer patients [11]. *Toxicodendron vernicifluum* (formerly *Rhus verniciflua*) Stokes belongs to the Anacardiaceae family and is commonly known as the lacquer tree. In East Asia, including Korea and China, it has long been used as a food supplement and traditional herbal medicine [12]. *Toxicodendron vernicifluum* Stokes (TVS) was found to contain a number of bioactive phytochemicals, including alkaloids, polyphenols and flavonoids [12]. Previous research has shown that TVS extract has antioxidant, anti-bacterial, anti-obesity, anti-proliferative, anti-inflammatory, and anti-tumor effects [12–22]. Despite the beneficial pharmacological effects of TVS, its use has been somewhat limited due to the presence of urushiol an allergenic substance, that is a mixture of several derivatives of catechol. In order to safely and more widely use TVS, it is essential to remove urushiol from extracts. Various methods have been developed to detoxification by removing urushiol from TVS [23–25]. Depending on the method, the main components, activities, and safety extracted from TVS differ. Therefore, it is important to find a safe way to selectively remove only urushiol and retain other healthy ingredients [12].

We applied a new variation to an existing extraction method to produce a new urushiol—free TVS extract (TVSE). Briefly, the method is a classic detoxification method that removes urushiol activity by high-temperature treatment in the process of hot water extraction of TVS, and adds a concentration and purification process. As a result of analyzing the new TVSE by HPLC, not only urushiol was not detected, but also the extract had a higher content of flavonoids such as fustin and fisetin than the existing method. We demonstrated *in vitro* studies that this new TVSE is safe and has anti-cancer effects [26].

This study was conducted to determine whether TVSE may help prevent breast cancer metastasis in an *in vivo* system. 4T1 murine mammary carcinoma cells were injected into the mammary fat pad of BALB/c mice to induce breast cancer, and then TVSE was orally administered to investigate the effects on solid tumor growth and cancer metastasis.

## Materials and methods

### Materials

Dulbecco’s Modified Eagle’s Medium (DMEM) and other cell culture reagents were purchased from Welgene (Daegu, Korea). Fetal bovine serum (FBS) was purchased from Cambrex Bio Technology (Walkersville, MD, USA). Antibodies against Ki67, platelet endothelial cell adhesion molecule (PECAM-1), and vascular endothelial growth factor (VEGF) were purchased from Cell Signal Technology (Beverly, MA, USA). Fluorochrome-conjugated secondary antibodies (Alexa-488 and 564) were purchased from Thermo Fisher Scientific (Waltham, MA, USA) while 4’,6-diamidino-2-phenylindole (DAPI) was purchased from Sigma-Aldrich (St. Louis, MO, USA).

### Preparation of TVSE

TVSE used in the experiment was prepared from Medience Co. Ltd, according to the methods described previously [26]. Dried trunk of TVS cultivated in Chuncheon, Korea were purchased from an herbal medicine store (Chuncheon, Korea). Dried trunk of TVS were sliced to 2 cm, after which, 100 g of sliced TVS was refluxed in 1 L of water at 100°C for 10 h. This extraction procedure was repeated twice. The extracts were filtered through Whatman filter paper #2, after which the filtrate was freeze-dried. To concentrate and refine, 100 g of freeze-dried powder was refluxed in 1L of 95% ethanol at room temperature for 1 h. The extract was centrifuged at 3,000 rpm for 10 min, after which the supernatant was collected and dried below 60°C in a vacuum. The resulting powder was used as TVSE. The contents of fustin and fisetin in TVSE were analyzed using an HPLC (SPD-20A, Shimadzu, Tokyo, Japan) as per method described previously [26]. The contents of fustin and fisetin were 219 mg/g of TVSE and 82 mg/g of TVSE, respectively.

### 4T1 cell culture

4T1 murine mammary carcinoma cells were acquired from the American Type Culture Collection (Rockville, MA, USA). 4T1 cells were cultured in DMEM supplemented with 100 mL/L fetal bovine serum, 100,000 U/L penicillin, and 100 mg/L streptomycin at 37°C in a humidified atmosphere of 5% CO_2_ and 95% air.

### Animals

All animal experiments were conducted according to protocols ratified by the Institutional Animal Care and Use Committee of Hallym University (Hallym 2018–22). Five-week-old female BALB/c mice were purchased from Dooyeol Biotech Co. Ltd. (Seoul, Korea) and housed at the animal research facility of Hallym University in controlled standard conditions: 23 ± 3°C temperature, 50 ± 10% relative humidity, and a 12-h light/dark cycle. The mice were fed on a commercial rodent diet (Cargil Agri Purina, Inc., Seongnam, Korea) and water provided *ad libitum*.

### *In vivo* orthotropic breast tumor experiment

After acclimation for 1 week, 4T1 cells (1 x 10^5^ cells) were injected into the mammary fat pads of mice. When tumors size reached a volume of 50 mm^3^, the mice were divided randomly into 5 groups: (1) 0 mg of TVSE/kg BW/day (vehicle fed, CON), (2) 50 mg of TVSE kg BW/day (T50), (3) 100 mg of TVSE/kg BW/day (T100), (4) 200 mg of TVSE/kg BW/day (T200), and (5) 400 mg of TVSE/ kg BW/day (T400). The mice were subjected to oral gavage with vehicle (distilled water) or TVSE (50, 100, 200, or 400 mg/kg BW/day) for 20 days. The tumor volume was measured with a set of calipers every 3 days and calculated using the formula 0.52 x long diameter x short dismeter^2^ [27]. After 20 days of administering TVSE, all mice were anesthetized with tribromoethanol diluted with tertiary amyl alcohol (1: 40 ratio), after which blood was collected from the orbital vein. The mice were euthanatized by carbon dioxide asphyxiation and the tumors, lungs, and livers were excised from the mice. The lungs were fixed in Bouin’s solution and the lung metastatic nodules were counted to evaluate metastasis. The tumors were formalin-fixed and paraffin-embedded for immunohistological analyses or homogenized to prepare total RNA for real-time RT-PCR.

### Immunofluorescence staining

Paraffin-embedded tumor tissues were sectioned to a thickness of 5 μm, deparaffinized with xylene, rehydrated through xylene and graded alcohol and blocked with 5% bovine serum albumin. Immunofluorescence (IF) staining was conducted with the indicated antibodies and fluorochrome-conjugated secondary antibodies (Alexa-488 and 564). Apoptotic cells were identified by terminal deoxynucleotidyl transfer-mediated dUTP nick-end labeling (TUNEL) staining using an *in situ* BrdU-Red DNA fragmentation assay kit (Abcam, Cambridge, UK). Nuclei were counterstained with DAPI. The slides were examined in a blinded manner and randomly chosen fields were photographed at 400x magnification. The immune-positive cells were quantified with a Carl Zeiss AxioImager microscope and Image M1 Software (Carl Zeiss, Jena, Germany).

### Quantitative real-time RT-PCR

Total RNA from the tumor tissue was extracted with Trizol (Invitrogen Life Technologies, Carlsbad, CA, USA) according to the manufacturer’s instruction. The content and the purity of the total RNA were determined using a micro-volume spectrophotometer (BioSpec-nano, Shimadzu, Kyoto, Japan). Complementary DNA was synthesized from 2 μg of total RNA using HyperScript^TM^ RT master mix kit (GeneAll Biotechnology, Seoul, Korea). Real-time PCR of cDNA was conducted using a Rotor-gene 3000 PCR (Corbett Research, Mortlake, Australia) and a Rotor-Gene^TM^ SYBR Green kit (Qiagen, Valencia, CA, USA) according to the manufacturer’s instructions. The primer sequences used in this study are shown in Table 1. The results were analyzed with Rotor-Gene 6000 Series System Software program, version 6 (Corbett Research) and normalized to those of glyceraldehyde 3-phosphate dehydrogenase (GAPDH).

**Table 1:** Primer sequences used in this study.

Target gene	Forward primer (5’-3’)	Reverse primer (5’-3’)
MMP-2	CCCATACTTTACTCGGA	TGACCTTGACCAGAACACCA
MMP-9	GTCTTCCTGGGCAAGCAGTA	CTGGACAGAAACCCCACTTC
TIMP-1	GGTTCCCTGGCATAATCTGA	GTCATCGAGACCCCAAGGTA
TIMP-2	GCATCACCCAGAAGAAGAGC	TGATGCAGGCAAAGAACTTG
uPA	GAAACCCTACAATGCCCACAGA	GACAAACTGCCTTAGGCCAATC
PAI-1	CCGTCTCTGTGCCCATGAT	GGCAGTTCCACGACGTCATA
ICAM-1	GTGGCGGGAAAGTTCCTG	CGTCTTGCAGGTCATCTTAGGAG
VCAM-1	AGTTGGGGATTCGGTTGTTC	CATTCCTTACCACCCCATTG
GAPDH	AGGTTGTCTCCTGCGACT	TGCTGTAGCCGTATTCATTGTCA

### Statistical analysis

All data are presented as the mean ± SEM and analyzed by analysis of variance. Differences between the treatment groups were analyzed by Duncan’s multiple range test. Differences were considered significant at *P* < 0.05. All statistical analyses were conducted using Statistical Analysis System for Windows version 9.4 (SAS Institute, Cary, NC, USA).

## Results

### TVSE inhibits solid tumor growth and lung metastasis of 4T1 cells in BALB/c mice

**Fig 1:**
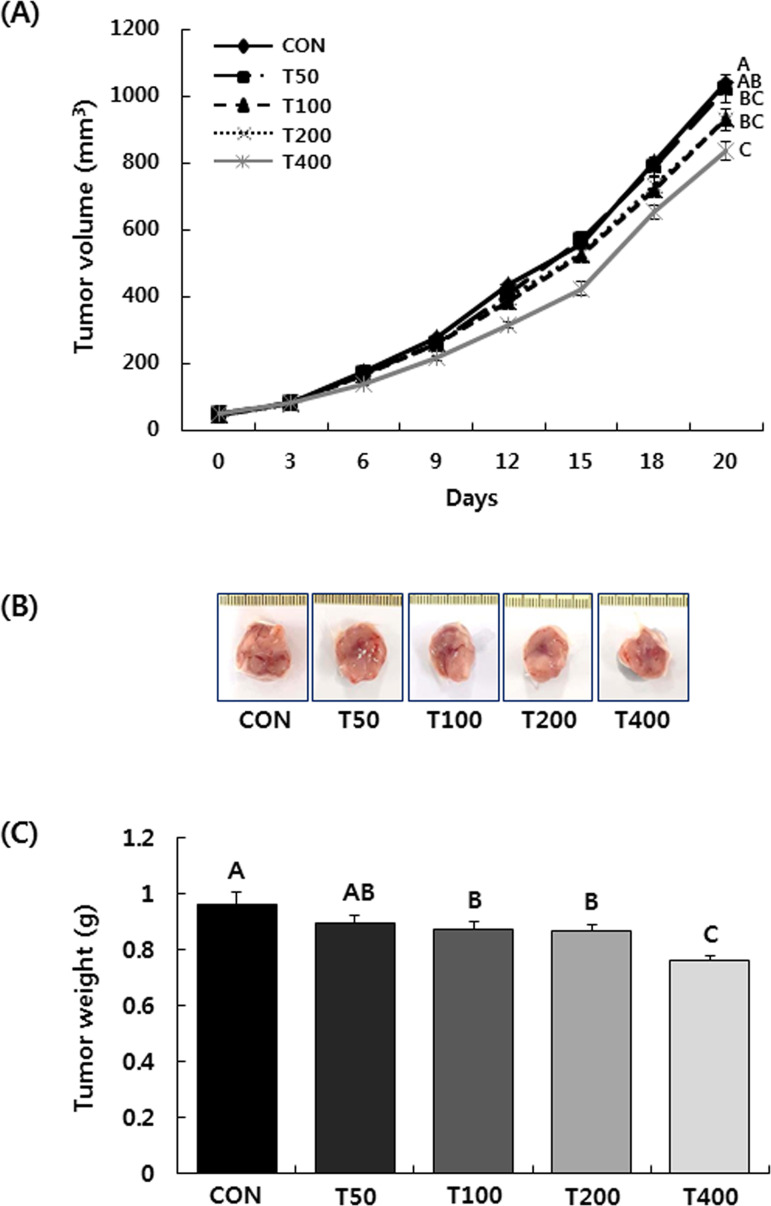
Oral administration of TVSE inhibits tumor growth in BALB/c mice injected with 4T1 mammary cancer cells. 4T1 cells (1 × 10^5^ cells/ mice) were injected into the mammary fat pads of six-week-old female BALB/c mice. After the tumor size reached 50 mm^3^ (7 days after 4T1 cell injection), TVSE was administered by oral gavage for 20 days. **(A)** The tumor volume was measured using calipers and calculated using the formula (0.52 × long diameter × short diameter^2^), n = 10. **(B)** At the end of the administration period, all mice were sacrificed. The tumors were excised and photographed. **(C)** Tumors weights. Each bar represents the mean ± SEM (n = 10). Means without a common letter differ, *P* < 0.05. CON, 0 mg of TVSE/kg body weight (BW)/day; T50, 50 mg of TVSE/ kg BW/day; T100, 100 mg of TVSE/ kg BW/day; T200, 200 mg of TVSE/ kg BW/day; T400, 400 mg of TVSE/ kg BW/day.

Seven days after the injection of 4T1 cells, solid tumors were visible and volumes were approximately 50 mm^3^. From then on, daily oral administration of TVSE was initiated, and continued for 20 days. The volume of tumors continued to increase until the mice were sacrificed. At the end of the experiment, the tumor volume in the CON group was 1,038 ± 26 mm^3^. The tumor volume in the T400 group was reduced by 19.5% compared to the CON group (Fig 1A). As shown in Fig 1B and 1C, the wet tumor weight was reduced dose-dependently by the administration of TVSE. The wet tumor weight in the T400 group was reduced by 20.9% compared to the CON group.

**Fig 2:**
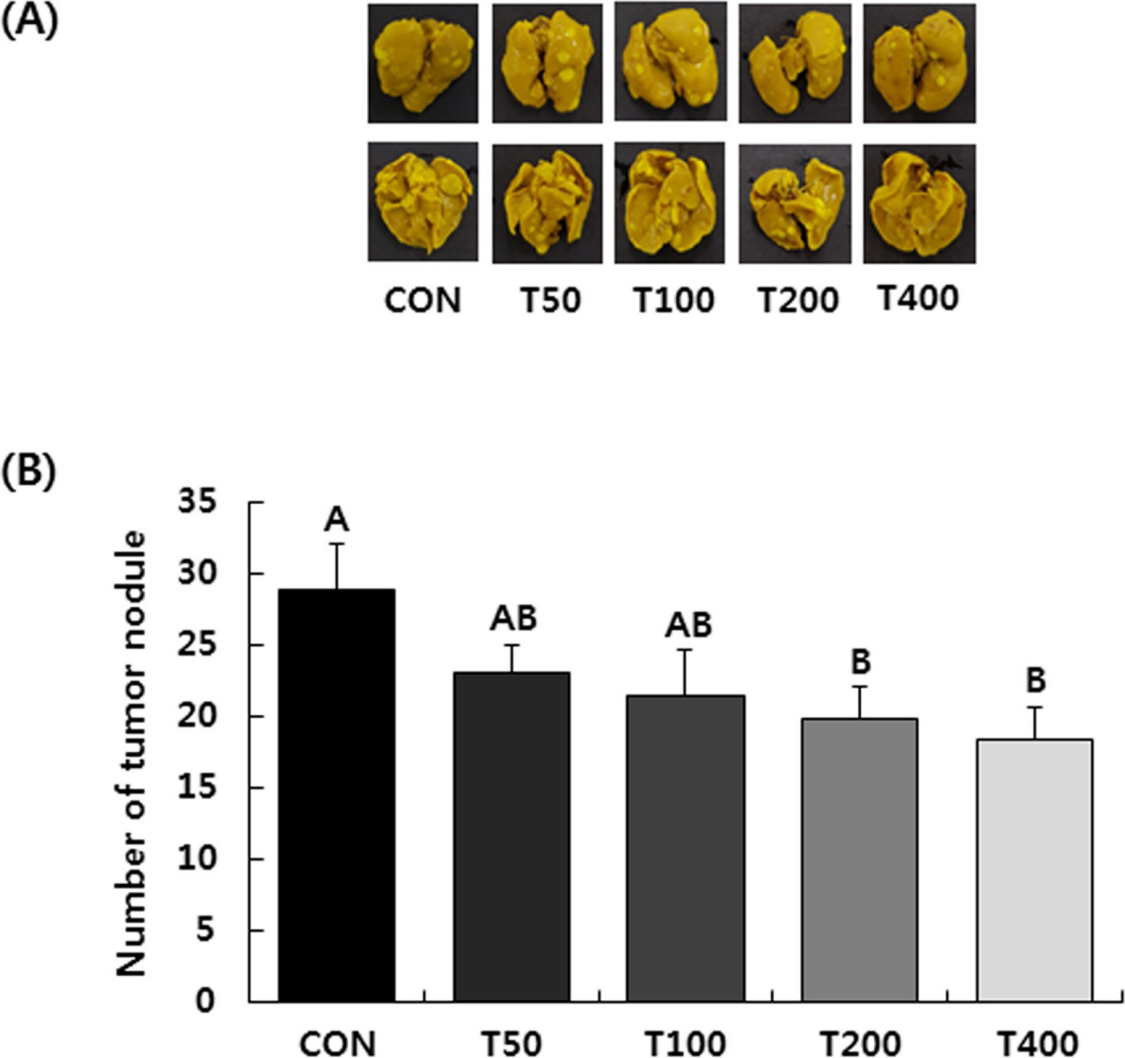
Oral administration of TVSE inhibits the lung metastasis of 4T1 cells in BALB/c mice. Mice were injected with 4T1 cells and administered with TVSE as described. After 20 days administration, the lungs were excised and fixed in Bouin’s solution. **(A)** Lung photographs. **(B)** Metastatic tumor nodules in the lungs were counted. Each bar represents the mean ± SEM (n = 10). Means without a common letter differ, *P* < 0.05. CON, 0 mg of TVSE/kg body weight (BW)/day; T50, 50 mg of TVSE/ kg BW/day; T100, 100 mg of TVSE/ kg BW/day; T200, 200 mg of TVSE/ kg BW/day; T400, 400 mg of TVSE/ kg BW/day.

Tumor nodules were observed in the lungs, but not in the livers when the mice were sacrificed 28 days after 4T1 cell injection. The administration of TVSE significantly reduced the number of tumor nodules on the lung, with the number in T50, T100, T200 and T400 groups reduced by 20.4, 26.0, 31.5 and 36.3%, respectively, compared to the CON group (Fig 2).

### TVSE inhibits cell proliferation and induces apoptosis in 4T1 tumors in BALB/c mice

**Fig 3:**
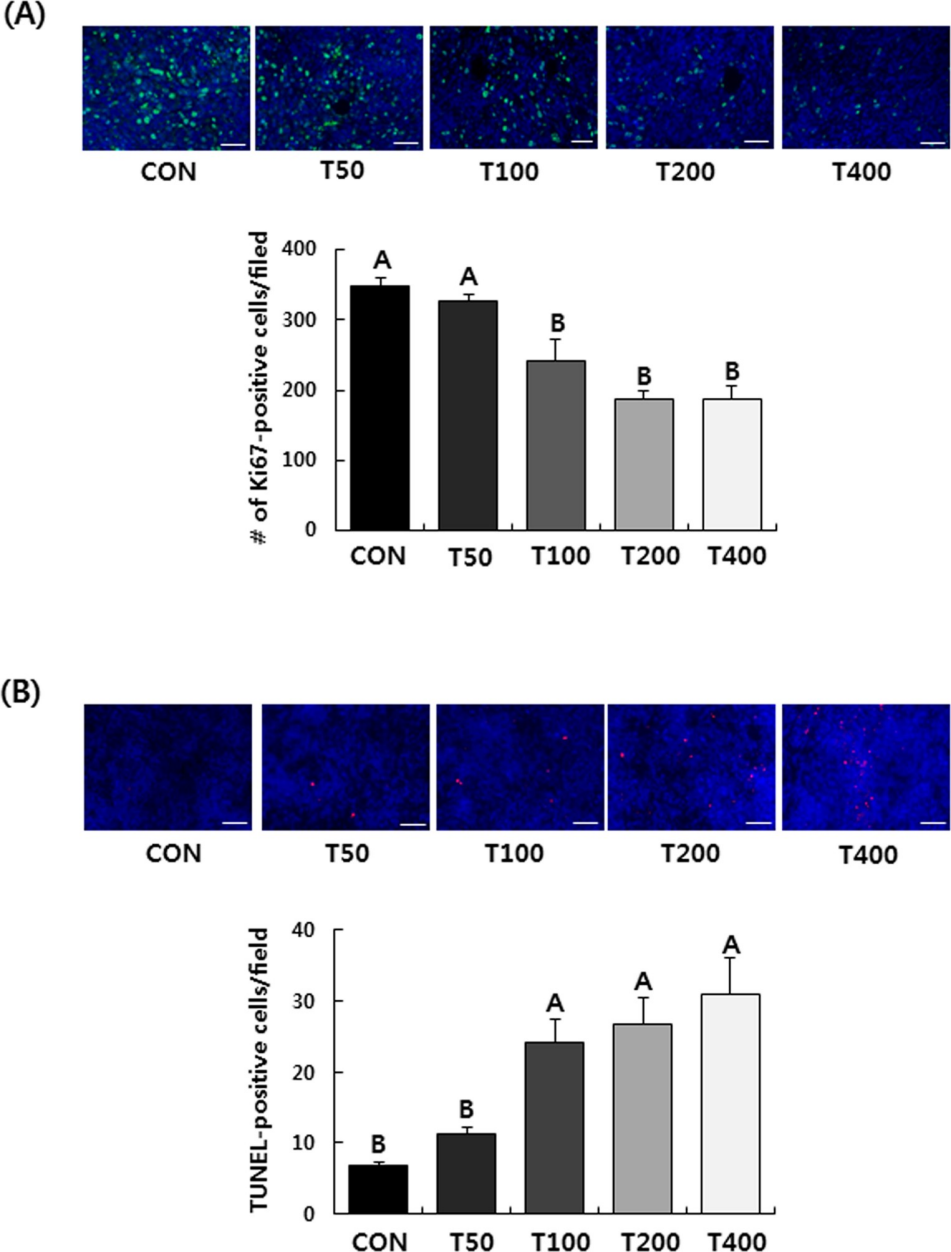
Oral administration of TVSE suppresses cell proliferation and induces apoptosis in 4T1 tumors in BALB/c mice. Mice were injected with 4T1 cells and administered with TVSE as described. **(A)** Tumor sections were stained with Ki67 antibody. Representative images of IF staining are shown. Scale bar, 100 μm (upper panel). The Ki67-positive cells were counted (lower panel). (**B**) Tumor sections were stained with TUNEL. Images of TUNEL staining are shown. Scale bar, 100 μm (upper panel). TUNEL-positive apoptotic cells were counted (lower panel). Each bar represents the mean ± SEM (n = 4). Means without a common letter differ significantly, *P* < 0.05. CON, 0 mg of TVSE/kg body weight (BW)/day; T50, 50 mg of TVSE/ kg BW/day; T100, 100 mg of TVSE/ kg BW/day; T200, 200 mg of TVSE/ kg BW/day; T400, 400 mg of TVSE/ kg BW/day.

IF staining results showed that the expression of Ki67, a cellular marker for proliferation, was noticeably suppressed in the tumor by the administration of TVSE (Fig 3A). As shown in Fig 3B, the number of TUNEL-positive apoptotic cells was markedly increased in tumors administered with TVSE (Fig 3B). In the T400 group, the expression of Ki67 was decreased by 46.1% but the number of TUNEL-positive apoptotic cells was increased by 456%, compared to the CON group (Fig 3).

### TVSE inhibits angiogenesis in 4T1 tumors in BALB/c mice

**Fig 4:**
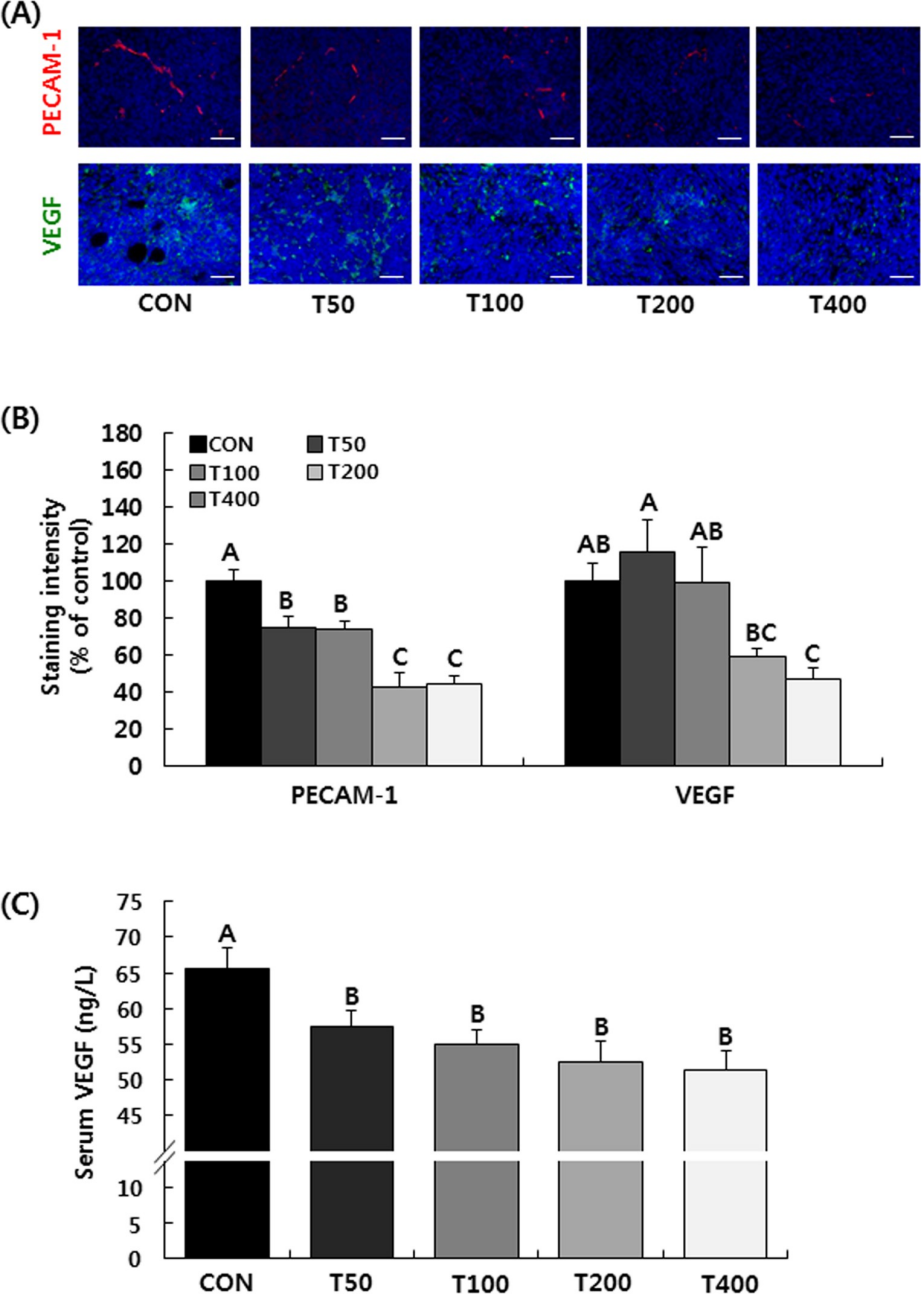
Oral administration of TVSE decreases angiogenesis in 4T1 tumors in BALB/c mice. Mice were injected with 4T1 cells and administered with TVSE as described. **(A)** Tumor sections were stained with PECAM-1 and VEGF antibodies. Representative images of IF staining are shown. Scale bar, 50 μm. **(B)** The staining intensity of the indicated protein was quantified. Each bar represents the mean ± SEM (n = 4). **(C)** Blood samples were collected from mice at sacrifice, and sera were prepared. Serum levels of VEGF were measured with a VEGF ELISA kit. Each bar represents the mean ± SEM (n = 10). Means without a common letter differ significantly, *P* < 0.05. CON, 0 mg of TVSE/kg body weight (BW)/day; T50, 50 mg of TVSE/ kg BW/day; T100, 100 mg of TVSE/ kg BW/day; T200, 200 mg of TVSE/ kg BW/day; T400, 400 mg of TVSE/ kg BW/day.

To evaluate the degree of tumor angiogenesis, the expression of PECAM-1, also known as cluster of differentiation 31 (CD31) in tumors was examined by IF staining. The expression of PECAM-1 was dose-dependently diminished by the administration of TVSE. In addition, the expression of VEGF in tumors was also significantly reduced by the administration of TVSE. Expression of PECAM-1 and VEGF were reduced by 55.5% and 52.6% in the T400 group, respectively, compared to the CON group (Fig 4A and 4B). As shown in Fig 4C, serum VEGF concentration was significantly reduced by the administration of TVSE, but there was no significant difference between the different groups administered TVSE.

### TVSE induces alternation in the transcript expression of metastasis-related genes in 4T1 tumors in BALB/c mice

**Fig 5:**
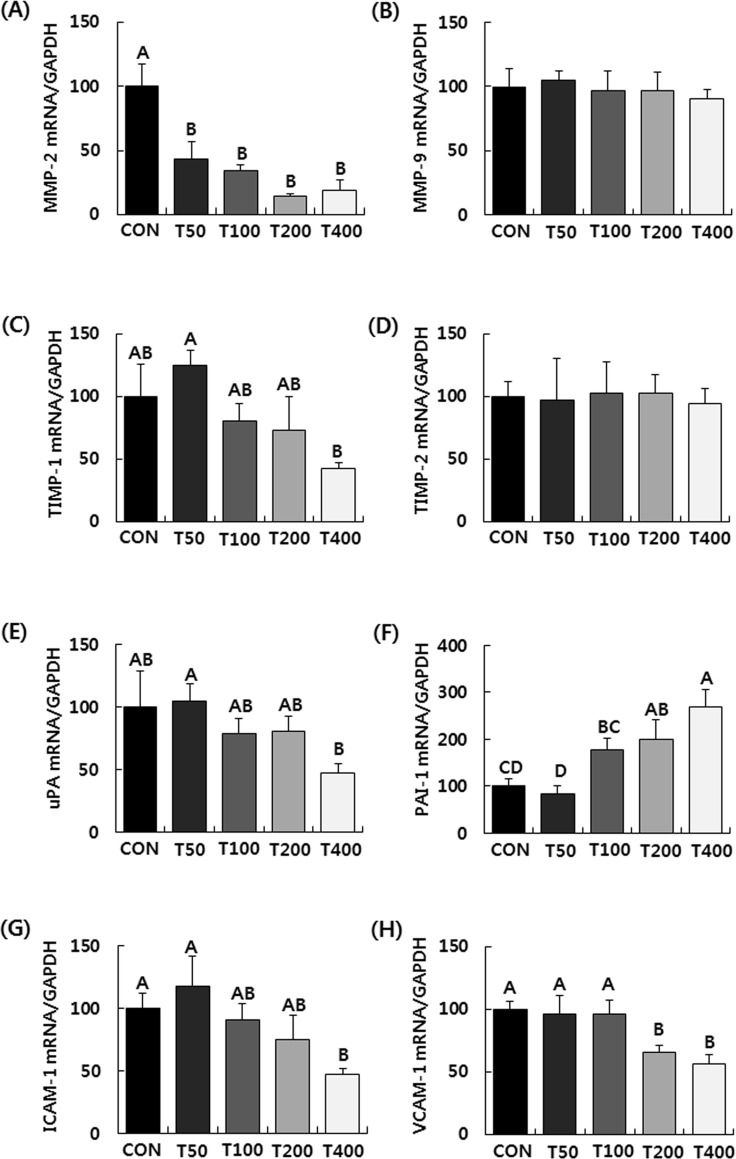
Oral administration of TVSE alters the mRNA expression of metastasis-related genes in 4T1 tumors in BALB/c mice. Mice were injected with 4T1 cells and administered with TVSE as described. The total RNA in tumors was extracted, reverse transcribed, and real-time PCR was conducted. The amount of each mRNA was normalized to the amount of glyceraldehyde 3-phosphate dehydrogenase (GAPDH) mRNA and the control levels were set at 100. Each bar represents the mean ± SEM (n = 10). Means without a common letter differ significantly, *P* < 0.05. CON, 0 mg of TVSE/kg body weight (BW)/day; T50, 50 mg of TVSE/ kg BW/day; T100, 100 mg of TVSE/ kg BW/day; T200, 200 mg of TVSE/ kg BW/day; T400, 400 mg of TVSE/ kg BW/day.

To elucidate the mechanisms by which TVSE inhibits lung metastasis, the mRNA expressions of metastasis-related genes were examined. The administration of TVSE significantly suppressed expression of matrix metalloproteinase (MMP)-2, tissue inhibitor of metalloproteinase (TIMP)-1, urokinase plasminogen activator (uPA), intercellular adhesion molecule (ICAM)-1, and vascular cell adhesion molecule (VCAM)-1 in tumors. Of these, MMP-2 mRNA expression was the most dramatically reduced and was 81.2% lower in the T400 group, compared to the CON group. In contrast, the administration with TVSE dramatically increased plasminogen activator inhibitor (PAI)-1 mRNA expression. Transcript expression of PAI-1 in the T400 group increased by 169.6% compared to the CON group. The administration of TVSE did not affect mRNA expression of MMP-9 and TIMP-2 in tumors (Fig 5).

## Discussion

Fustin and fisetin both have antioxidant properties [28, 29]. According to a recent comparative analysis of antioxidant activity of substances in *Rhus typhina* L. stem, fustin is less than methyl gallate, gallic acid and quercetin, but has a greater antioxidant capacity than vitamin C and rutin [30]. Fisetin from TVS significantly reduced inflammatory cytokines [31]. In *in vitro* studies, it has been reported that fisetin has anti-cancer effects by affecting various cell signal pathways and transcriptional factors; fisetin inhibited cancer cell proliferation by down-regulating AMP-activated protein kinase, cyclooxygensae, epidermal growth factor receptor, extracellular signal regulated kinase 1/2, MMP, nuclear factor-kappa B, prostate-specific antigen, transcription factor T-cell factor, TNF-related apoptosis inducing ligand, Wnt inhibitory factor, X linked inhibitor of apoptosis, and PI3K/AKT/mTOR pathway [32–36]. Fisetin also showed anti-cancer effects in cells and animal models by influencing cell cycles [37] and acting as a topoisomerase inhibitor [38].

In this study, after inducing tumors by injection of 4T1 murine mammary carcinoma cells into BALB/c mice, oral administration of TVSE for 20 days resulted in dose-dependent tumor growth inhibition. In the T400 group, tumor volume and weight were reduced by 19.5% and 20.9%, respectively, compared to the control group (Fig 1A). Ki67 expression and TUNEL-positive cell analysis showed that at doses above TVSE 100 mg/kg BW/day, cancer cell proliferation was significantly reduced and apoptosis was increased (Fig 3A and 3B). These results show that TVSE inhibits the solid tumor growth of breast cancer.

On day 28 of 4T1 cell injection, several tumor nodules were found in the lungs of BALB/c mice, indicating metastasis to lungs. However, in mice treated with TVSE, the number of tumor nodules was reduced in a dose-dependent manner (Fig 2B). The potential for primary tumor cells becoming macrometastases at secondary sites is a very inefficient process (0.01%) [39]. Long ago, it was suggested that cancer metastasis follows a pattern of organ-specific metastasis rather than randomness, which is influenced by the microenvironment of secondary organs [40]. This is supported by the creation of tissue media in areas where breast cancer metastasizes well in general (lymph node, lung, liver, bone, brain), showing that breast cancer cells exhibit organ-specific responses in proliferation and migration [41]. Breast cancer spreads particularly well to bones, lungs, liver and brain. In breast cancer patients with metastases; 30–60% occurs in bones, 4–10% in brains, 15–32% in livers, and 21–32% metastases have been reported in the lungs [9], which is explained by the fact that certain organs not only provide the physical environment in which breast cancer metastasis occurs, but also produce soluble components that facilitate growth.

The lungs are the major capillary bed where breast cancer cells meet for the first time after they exit the bloodstream. The capillaries of the lungs are 1/5 the size of the tumor cells, so breast cancer cells in the capillaries are more likely to arrest. In addition, successful metastasis followed by the occurrence of transendothelial migration and extravasation is regulated by the expression of cell surface markers specific to the lung microenvironment [42, 43], various tumor secretion factors, exosomes, and substrate components [10]. Lung metastasis tends to occur, especially within five years after initial breast cancer diagnosis, and has a significant impact on morbidity and mortality in patients. The average time of survival after breast cancer metastasis to the lung is only 22 months [44]. It is estimated that 60 to 70% of patients who die from breast cancer have lung metastases [45]. Therefore, in breast cancer patients, inhibiting metastasis to the lung is expected to have a significant effect on the survival rate. In the current study, tumor nodules were found only in lung, but not in liver. Nonetheless, it is extremely meaningful that metastasis to the lung can be significantly reduced by TVSE administration.

To begin to define the mechanism by which TVSE suppresses lung metastasis in breast cancer, we identified the expression of genes associated with metastasis: MMP-2, MMP-9, TIMP-1, TIMP-2, uPA, PAI-1, ICAM-1, and VCAM-1. RVSE administration decreased MMP-2, uPA, ICAM-1, and VCAM-1, but increased PAI-1. The gene expression of TIMP-1 and TIMP-2 showed differences in response to administration of TVSE. While TIMP-2 was not significantly different between control and TVSE administration groups, TIMP-1 tended to decrease with increasing TVSE administration. Unlike MMP-2, MMP-9 was not affected by TVSE administration (Fig 5).

MMP family proteins are involved in breaking down the ECM during normal physiological processes such as embryonic development, reproduction, and tissue regeneration as well as during disease processes such as arthritis and cancer. In cancer progression, MMPs break down the ECM allowing cancer cells to migrate out of the primary tumor and metastasize. MMP-2, along with MMP-9, degrades type IV collagen, the most abundant component of the basement membrane. Since the basement membrane plays important roles in the maintenance of tissue composition, in providing structural support, and in maintaining cellular signaling and polarity, its breakdown is an essential step in the metastatic progression of most cancers [46]. MMP-2 and MMP-9 also play important roles in the angiogenesis and lymphangiogenesis required for metastasis [47]. In particular, MMP-2 may affect tumor growth, invasion and metastasis by regulating lymphatic vessel formation as well as angiogenesis [48]. In this study, TVSE reduced MMP-2 gene expression, but did not affect MMP-9. The mechanism behind this differential responsiveness requires further study.

TIMP-1 and TIMP-2 are natural inhibitors of MMPs. We anticipated that the expression of TIMP-1 and TIMP-2 would be elevated by TVSE supplementation, but TIMP-1 tended to decrease dose-dependently in RVSE, and there was no change in TIMP-2. TVSE therefore seems to have relatively little effect on these genes of proteins that act as metalloproteinase suppressors. On the other hand, it has been reported that the incidence of cancer is increased when the actions of TIMP-1 and TIMP-2 are abnormal, and increased expression of TIMP-1 has been suggested to be a useful basis for diagnosing of malignant tumors [49, 50]. In this respect, the reduction of TIMP-1 gene expression by TVSE may be considered a positive influencer of cancer growth and metastasis. However, as with MMP-2 and MMP-9, the different results between TIMP-1 and TIMP-2, suggest further study is needed.

uPA is a serine protease involved in degradation of the extracellular matrix and possibly tumor cell migration and proliferation. ICAM-1 is a type of epithelial and leukocyte-associated transmembrane protein that promotes leukocyte endothelial transmigration [51]. When ICAM-1 is expressed in vascular endothelial cells, it binds to the integrin LFA-1, a receptor found on leukocytes. And activated leukocytes bind to endothelial cells via ICAM-1/LFA-1 and then transmigrate into tissue [52]. VCAM-1 mediates the attachment of lymphocytes, monocytes, eosinophils, and basophils to vascular epithelial cells [53]. PAI-1 is the most important inhibitor of uPA, and irreversibly inhibits its proteolytic activity [54]. PAI-1 inhibits uPA through active site binding and inhibits fibrin degradation, a physiological process that prevents the formation of plasmin and lowers blood clots. PAI-1 also inhibits the activity of MMPs, which play a critical role in the invasion of malignant cells through the basal layer. Increases in PAI-1 have been found in several forms of cancer, and also in obesity and metabolic syndrome which are associated with increased thrombosis [55]. In this study, the T400 group showed significantly lower gene expression of uPA, ICAM-1, and VCAM-1, and PAI-1 was significantly higher than in other experimental groups. These indicate that TVSE reduces gene transcription related to angiogenesis and lymphangiogenesis, processes related to promotion of metastasis and simultaneously increases gene expression associated with inhibition of metastasis.

Solid tumors cannot grow without a proper blood supply. This implies that angiogenesis must accompany the early stages of metastasis. We examined the effect of TVSE on angiogenesis by analyzing expression of PECAM-1 and VEGF in tumor tissues. PECAM-1 is found on the surface of platelets, monocytes, neutrophils, and T-cells and is present in a substantial portion of endothelial cell intercellular junctions. It is involved in leukocyte migration, angiogenesis and integrin activation. The expression of PECAM-1 in tissues can be applied to assess the extent of tumor angiogenesis [56]. VEGF, originally known as vascular permeability factor, is a signaling protein produced by cells that stimulates the formation of blood vessels [57]. VEGF is responsible for fetal development and creation of new blood vessels after wounding or to bypass blocked vessels. However, when VEGF is overexpressed, it can contribute to disease. Cancer cells can facilitate growth and metastasis by expressing VGEF. Numerous studies have shown reduced overall and disease-free survival in tumors that overexpress VEGF [57]. In particular, VEGF is associated with a poor prognosis in breast cancer. Overexpression of VEGF corresponds to an angiogenic switch. Increased blood VEGF levels have been reported in angiosarcoma patients [58]. Once released, VEGF induces several responses, including cell survival, migration, and further differentiation. Therefore, the use of VEGF as a target in cancer treatment was explored and in 2004, the first anti-VEGF drug, bevacizumab, was approved. In the current study, oral administration of TVSE between 50 and 400 mg/kg BW/day resulted in dose-dependent decreases in PECAM-1 and VEGF (Fig 4A and 4B). Serum VEGF levels were significantly decreased by TVSE administration, but the same results were observed at all concentrations (Fig 4C). These results show that even low levels of TVSE administration can effectively prevent angiogenesis. TVS has been reported to increase the survival rate and slow the progression of cancer by applying it to patients with metastasized end-stage colon or pancreatic cancer and non-small cell lung cancer [59–62]. These results show that TVSE is likely to be used as a substitute for overcoming the side effects of chemotherapeutic agents in drug-resistant cancer including breast cancer. Perhaps the combination of chemotherapeutic drugs and TVSE can create a more synergistic effect. Further research on this is needed in the future.

In Korea, TVS extract or powder has long been traditionally used for food or medicinal purposes. In recent years, TVSE powder with no urushiol detected and fustin (an indicator) content of 57 mg/kg has been certified as a health functional food material (which may help men's health in menopause) [63]. This is because toxicity, side effects, and adverse reactions have not been confirmed, and its function as well as safety have been confirmed in human application tests conducted with the same raw materials. Its daily intake was 1 g/day. The TVSE concentration of 50–400 mg/kg which was used on experimental animals in this study is converted in terms of the human body to a high concentration of 0.25–2.0 g. Although human studies have reported that TVSE 120 mg/kg body weight was safe [64], further studies are needed to determine whether it is safe at higher doses as well.

## Conclusion

This study was performed to investigate the effects of TVSE produced by a new extraction method, on the growth and lung metastasis of breast cancer *in vivo*. TVSE dose-dependently decreased breast tumor volume and weight and the number of lung tumor nodules. Expression of Ki67 was reduced and the number of apoptotic cells increased by TVSE administration. PECAM-1 and VEGF levels in breast tumor cells were reduced by TVSE administration. TVSE decreased gene expression of MMP-2, TIMP-1, uPA, ICAM-1, and VCAM-1 and increased that of PAI-1. Collectively, these results indicate that TVSE inhibits both metastasis to the lung as well as growth of solid tumors in the breast. Mechanistically, TVSE affects the transcriptional regulation of several genes involved in metastasis and the data suggests that TVSE could be used as an effective alternative or supplement for breast cancer treatment and metastatic inhibition. Further research is needed to investigate the effects of TVSE on different types of breast cancer, and to determine whether TVSE is effective in inhibiting metastasis in other organs that are specific for breast cancer metastasis. Detailed mechanisms involved in inhibiting metastasis also require further study.

## References

[1] Bray F, Ferlay J, Soerjomataram I, Siegel RL, Torre LA, Jemal A. Global cancer statistics 2018: GLOBOCAN estimates of incidence and mortality worldwide for 36 cancers in 185 countries. CA Cancer J Clin. 2018; 68:394–424. 10.3322/caac.21492 30207593

[2] Jin L, Han B, Siegel E, Cui Y, Giuliano A, Cui X. Breast cancer lung metastasis: Molecular biology and therapeutic implications. Cancer Biol Ther. 2018;19:858–68. 10.1080/15384047.2018.1456599 29580128 PMC6300341

[3] Chaffer CL, Weinberg RA. A perspective on cancer cell metastasis. Science. 2011;331:1559–64. 10.1126/science.1203543 21436443

[4] Eckhardt BL, Francis PA, Parker BS, Anderson RL. Strategies for the discovery and development of therapies for metastatic breast cancer. Nat Rev Drug Discov. 2012;11:479–97. 10.1038/nrd2372 22653217

[5] Gligorov J, Lotz JP. Optimal treatment strategies in postmenopausal women with hormone-receptor-positive and HER2-negative metastatic breast cancer. Breast Cancer Res Treat. 2008; 112 Suppl 1:53–66. 10.1007/s10549-008-0232-x 19101794

[6] Chambers AF, Groom AC, MacDonald IC. Dissemination and growth of cancer cells in metastatic sites. Nat Rev Cancer. 2002; 2:563–72. 10.1038/nrc865 12154349

[7] Polyak K, Kalluri R. The role of the microenvironment in mammary gland development and cancer. Cold Spring Harb Perspect Biol. 2010;2:a003244 10.1101/cshperspect.a003244 20591988 PMC2964182

[8] Perou CM, Sørlie T, Eisen MB, van de Rijn M, Jeffrey SS, Rees CA, et al Molecular portraits of human breast tumours. Nature. 2000;406:747–52. Available from: 10.1038/35021093 10963602

[9] Wu Q, Li J, Zhu S, Wu J, Chen C, Liu Q, et al Breast cancer subtypes predict the preferential site of distant metastases: a SEER based study. Oncotarget. 2017;8:27990–6. 10.18632/oncotarget.15856 28427196 PMC5438624

[10] Medeiros B, Allan AL. Molecular mechanisms of breast cancer metastasis to the lung: Clinical and experimental perspectives. Int J Mol Sci. 2019;20:2272 10.3390/ijms20092272 31071959 PMC6540248

[11] Qamar H, Rehman S, Chauhan DK. Current status and future perspective for research on medicinal plants with anticancerous activity and minimum cytotoxic value. Current Drug Targets 2019; 20: 1227–43. 10.2174/1389450120666190429120314 31486747

[12] Lee SO, Kim SJ, Kim JS, Ji H, Lee EO, Lee HJ. Comparison of the main components and bioactivity of *Rhus verniciflua* Stokes extracts by different detoxification processing methods. BMC Complement Altern Med. 2018;18:242 10.1186/s12906-018-2310-x 30165848 PMC6118002

[13] Jang IS, Park JW, Jo EB, Cho CK, Lee YW, Yoo HS, et al Growth inhibitory and apoptosis-inducing effects of allergen-free *Rhus verniciflua* Stokes extract on A549 human lung cancer cells. Oncol Rep. 2016;36: 3037–43. 10.3892/or.2016.5131 27667098

[14] Jung CH, Jun CY, Lee S, Park CH, Cho K, Ko SG. *Rhus verniciflua* stokes extract: radical scavenging activities and protective effects on H_2_O_2_-induced cytotoxicity in macrophage RAW 264.7 cell lines. Biol Pharm Bull. 2006;29:1603–7. 10.1248/bpb.29.1603 16880612

[15] Jung CH, Kim JH, Hong MH, Seog HM, Oh SH, Lee PJ, et al Phenolic-rich fraction from Rhus verniciflua Stokes (RVS) suppress inflammatory response via NF-kappaB and JNK pathway in lipopolysaccharide-induced RAW 264.7 macrophages. J Ethnopharmacol. 2007; 110:490–7. 10.1016/j.jep.2006.10.013 17112694

[16] Kim MS, Lee CW, Kim JH, Lee JC, An WG. Extract of *Rhus verniciflua* Stokes induces p53-mediated apoptosis in MCF-7 breast cancer cells. Evid Based Complement Alternat Med. 2019; 9407340 Available from: 10.1155/2019/9407340 30881477 PMC6383427

[17] Li W, Kim TI, Kim JH, Chung H-S. Immune checkpoint PD-1/PD-L1 CTLA-4/CD80 are blocked by *Rhus verniciflua* Stokes and its active compounds. Molecules. 2019;24:4062 10.3390/molecules24224062 31717574 PMC6891444

[18] Saravanakumar K, Chelliah R, Hu X, Oh DH, Kathiresan K, Wang MH. Antioxidant, anti-lung cancer, and anti-bacterial activities of toxicodendron vernicifluum. Biomolecules. 2019; 9:127 Available from: 10.3390/biom9040127 PMC6523688 30934938

[19] Suruga K, Tomita T, Kadokura K, Arai T. *Rhus verniciflua* leaf extract suppresses obesity in high-fat diet-induced obese mice. Food Nutr Res. 2019, 63 Available from: 10.29219/fnr.v63.3601 31548839 PMC6744841

[20] Lee KW, Um ES, Jung BB, Choi ES, Kim EY, Lee S, et al *Rhus verniciflua* Stokes extract induces inhibition of cell growth and apoptosis in human chronic myelogenous leukemia K562 cells. Oncol Rep. 2018; 39:1141–7. 10.3892/or.2018.6179 29328387

[21] Kang SH, Hwang IH, Son E, Cho CK, Choi JS, Park SJ, et al Allergen-removed *Rhus verniciflua* extract induces ovarian cancer cell death via JNK activation. Am J Chin Med. 2016;44:1719–35. Available from: 10.1142/S0192415X16500968 27848251

[22] Kim JH, Kim HP, Jung CH, Hong MH, Hong MC, Bae HS, et al Inhibition of cell cycle progression via p27Kip1 upregulation and apoptosis induction by an ethanol extract of *Rhus verniciflua* Stokes in AGS gastric cancer cells. Int J Mol Med. 2006;8:201–8. Available from: 10.3892/ijmm.18.1.201 16786174

[23] Choi HS, Yeo SH, Jeong ST, Choi JH, Park HS, Kim MK. Preparation and characterization of urushiol free fermented *Rhus verniciflua* stem bark (FRVSB) extracts. Korean J Food Sci Technol. 2012;44:173–8. 10.9721/KJFST.2012.44.2.173

[24] Kobayashi S, Ikeda R, Oyabu H, Tanaka H, Uyama H. Artificial Urushi: design, synthesis, and enzymatic curing of new urushiol analogues. Chem Lett. 2000;29:1214–5. Available from: 10.1246/cl.2000.1214

[25] Choi HS, Kim MK, Park HS, Yun SE, Mun SP, Kim JS, et al Biological detoxification of lacquer tree (*Rhus verniciflua* Stokes) stem bark by mushroom species. Food Sci Biotechnol. 2007;16:935–42. Available from: https://www.earticle.net/Article/A79270

[26] Lee HS, Jung JI, Kim KH, Park SJ, Kim EJ. *Rhus verniciflua* Stokes extract suppresses migration and invasion in human gastric adenocarcinoma AGS cells. Nutr Res & Prac. 2020 (In press). 10.4162/nrp.2020.14.5.463 33029287 PMC7520559

[27] Sauter BV, Martinet O, Zhang WJ, Mandeli J, Woo SL. Adenovirus-mediated gene transfer of endostatin in vivo results in high level of transgene expression and inhibition of tumor growth and metastases. Proc Natl Acad Sci U.S.A. 2000;97:4802–7. 10.1073/pnas.090065597 10758166 PMC18313

[28] Park BC, Lee YS, Park HJ, Kwak MK, Yoo KY, Kim JA. Protective effects of fustin, a flavonoids *Rhus verniciflua* Stokes, on 6-hydroxydopamine-induced neuronal cell death. Experimental & Molecular Medicine. 2007;39(3):316 Available from: 10.1038/emm.2007.35 17603285

[29] Grynkiewicz G, Demchuk OM. New perspectives for fisetin. Frontiers in Chemistry. 2019;7:697 10.3389/fchem.2019.00697 31750288 PMC6842927

[30] Liu T, Li Z, Li R, Cui Y, Zhao Y, Yu Z. Composition analysis and antioxidant activities of the *Rhus typhina* L. stem. J Pharmaceutical Analysis. 2019;9:332–8. Available from: 10.1016/j.jpha.2019.01.002 31929942 PMC6951479

[31] Lee JD, Huh JE, Jeon G, Yang HR, Woo HS, Choi DY, et al Flavonol-rich RVHxR from *Rhus verniciflua* Stokes and its major compound fisetin inhibits inflammation-related cytokines and angiogenic factor in rheumatoid arthritic fibroblast-like synovial cells and in vivo models. Int Immunopharmacol. 2009;9:268–76. 10.1016/j.intimp.2008.11.005 19111632

[32] Lall RK, Adhami VM, Mukhtar H. Dietary flavonoid fisetin for cancer prevention and treatment. Mol Nutr Food Res. 2016;60:1396–1405. 10.1002/mnfr.201600025 27059089 PMC6261287

[33] Hostetler GL, Ralston RA, Schwartz SJ. Flavones: food sources, bioavailability, metabolism, and bioactivity. Adv Nutr. 2017;8:423–35. Available from: 10.3945/an.116.012948 28507008 PMC5421117

[34] Kashyap D, Sharma A, Sak K, Tuli HS, Buttar HS, Bishayee A. Fisetin: a bioactive phytochemical with potential for cancer prevention and pharmacotherapy. Life Sci. 2018;194:75–87. 10.1016/j.lfs.2017.12.005 29225112

[35] Wang TY, Li Q, Bi K. Bioactive flavonoids in medicinal plants: structure, activity and biological fate. Asian J Pharm Sci. 2018;13:12–23. Available from: 10.1016/j.ajps.2017.08.004 32104374 PMC7032191

[36] Syed DN, Adhami VM, Khan MI, Mukhtar H. Inhibition of Akt/mTOR signaling by the dietary flavonoid fisetin. Anticancer Agents Med Chem. 2013;13(7):995–1001 10.2174/18715206113139990129 23293889 PMC3985520

[37] Gupta SC, Tyagi AK, Deshmukh-Taskar P, Hinojosa M, Prasad S, Aggarwal BB. Downregulation of tumor necrosis factor and other proinflammatory biomarkers by polyphenols. Arch Biochem Biophys. 2014;559: 91–9. 10.1016/j.abb.2014.06.006 24946050

[38] Salerno S, Da Settimo F, Taliani S, Simorini F, La Motta C, Fornaciari G, et al Recent advances in the development of dual topoisomerase I and II inhibitors as anticancer drugs. Curr Med Chem. 2010;17(35):4270–90. 10.2174/092986710793361252 20939813

[39] Luzzi KJ, MacDonald IC, Schmidt EE, Kerkvliet N, Morris VL, Chambers AF, et al Multistep nature of metastatic inefficiency: dormancy of solitary cells after successful extravasation and limited survival of early micrometastases. Am J Pathol. 1998;153: 865–73. 10.1016/S0002-9440(10)65628-3 9736035 PMC1853000

[40] Paget S. The distribution of secondary growths in cancer of the breast. Cancer Metastasis Rev. 1889;3421:571–3. Available from: 10.1016/S0140-6736(00)49915-0 2673568

[41] Chu JE, Xia Y, Chin-Yee B, Goodale D, Croker AK, Allan AL. Lung-derived factors mediate breast cancer cell migration through CD44 receptor-ligand interactions in a novel ex vivo system for analysis of organ-specific soluble proteins. Neoplasia. 2014;16: 180–91. 10.1593/neo.132076 24709425 PMC3978398

[42] MacDonald IC, Groom AC, Chambers AF. Cancer spread and micrometastasis development: quantitative approaches for in vivo models. Bioassays. 2002;24:885–93. 10.1002/bies.10156 12325121

[43] Stott SL, Hsu C-H, Tsukrov DI, Yu M, Miyamoto DT, Waltman BA, et al Isolation of circulating tumor cells using a microvortex-generating herringbone-chip. Proc Natl Acad Sci U.S.A. 2010;107:18392–7. 10.1073/pnas.1012539107 20930119 PMC2972993

[44] Smid M, Wang Y, Zhang Y, Sieuwerts AM, Yu J, Klijn JGM, et al Subtypes of breast cancer show preferential site of relapse. Cancer Res. 2008;68:3108–14. 10.1158/0008-5472.CAN-07-5644 18451135

[45] Schlappack OK, Baur M, Steger G, Dittrich C, Moser K. The clinical course of lung metastases from breast cancer. Klin Wochenschr. 1988;66:790–5. Available from: 10.1007/BF01726581 3184763

[46] Wertheim KY, Roose T. A mathematical model of lymphangiogenesis in a zebrafish embryo. Bull Math Biol. 2017;79: 693–737. 10.1007/s11538-017-0248-7 28233173 PMC5501200

[47] Mook ORF, Frederiks WM, Van Noorden CJF. The role of gelatinases in colorectal cancer progression and metastasis. Biochim Biophys Acta. 2004;1705:69–89. Available from: 10.1016/j.bbcan.2004.09.006 15588763

[48] Groblewska M, Siewko M, Mroczko B, Szmitkowski M. The role of matrix metalloproteinases (MMPs) and their inhibitors (TIMPs) in the development of esophageal cancer. Folia Histochem Cytobiol. 2012;50:12–19. 10.2478/18691 22532131

[49] Tarhini AA, Lin Y, Yeku O, LaFramboise WA, Ashraf M, Sander C, et al A four-marker signature of TNF-RII, TGF-α, TIMP-1 and CRP is prognostic of worse survival in high-risk surgically resected melanoma. J Transl Med. 2014;12:19 10.1186/1479-5876-12-19 24457057 PMC3909384

[50] Kim YS, Kim SH, Kang JG, Ko JH. Expression level and glycan dynamics determine the net effects of TIMP-1 on cancer progression. BMB Rep. 2012;45:623–8. 10.5483/bmbrep.2012.45.11.233 23187000 PMC4133808

[51] Yang L, Froio RM, Sciuto TE, Dvorak AM, Alon R, Luscinskas FW. ICAM-1 regulates neutrophil adhesion and transcellular migration of TNF-alpha-activated vascular endothelium under flow. Blood. 2005;106:584–92. 10.1182/blood-2004-12-4942 15811956 PMC1635241

[52] Xiao X, Mruk DD, Cheng CY. Intercellular adhesion molecules (ICAMs) and spermatogenesis. Hum Reprod Update. 2013;19:167–86. 10.1093/humupd/dms049 23287428 PMC3576004

[53] Cybulsky MI, Fries JW, Williams AJ, Sultan P, Davis VM, Gimbrone MA, et al Alternative splicing of human VCAM-1 in activated vascular endothelium. Am J Pathol. 1991;138: 815–20 1707234 PMC1886101

[54] Degryse B. The urokinase receptor system as strategic therapeutic target: challenges for the 21st century. Curr Pharm Des. 2011;17:1872–3. 10.2174/138161211796718161 21711231

[55] Vaughan DE. PAI-1 and atherothrombosis. J Thromb Haemost. 2005;3:1879–83. 10.1111/j.1538-7836.2005.01420.x 16102055

[56] Li G, Han ZL, Dong HG, Zhang X, Kong XQ, Jin X. Platelet endothelial cell adhesion molecule-1 gene 125C/G polymorphism is associated with deep vein thrombosis. Mol Med Rep. 2015;12: 2203–10. 10.3892/mmr.2015.3586 25846278

[57] Shibuya M. Vascular endothelial growth factor (VEGF) and its receptor (VEGFR) signaling in angiogenesis: a crucial target for anti- and pro-angiogenic therapies. Genes Cancer. 2011; 2:1097–105. 10.1177/1947601911423031 22866201 PMC3411125

[58] Melincovici CS, Boşca AB, Şuşman S, Mărginean M, Mihu C, Istrate M, et al Vascular endothelial growth factor (VEGF)—key factor in normal and pathological angiogenesis. Rom J Morphol Embryol. 2018;59:455–67. 10.1158/0008-5472.CAN-07-5809 30173249

[59] Lee SH, Choi WC, Yoon SW. Impact of standardized *Rhus verniciflua* stokes extract as complementary therapy on metastatic colorectal cancer: a Korean single-center experience. Integr Cancer Ther, 2009:8: 148–52. 10.1177/1534735409336438 19679623

[60] Lee SH, Kim KS, Choi WC, Yoon SW. The concurrent use of *Rhus verniciflua* stokes as complementary therapy with second or more line regimen on advanced non-small-cell lung cancer: case series. The Journal of Korean Oriental Medicine 2009;30:112–7. Available from: https://kmbase.medric.or.kr/Main.aspx?d=KMBASE&i=0363620090300060112&m=VIEW

[61] Lee S, Kim K, Jung H, Lee S, Cheon S, Kim S, et al Efficacy and safety of standardized allergen-removed *Rhus verniciflua* Stokes extract in patients with advanced or metastatic pancreatic cancer: a Korean single-center experience. Oncol. 2011;81:312–8. 10.1159/000334695 22179506

[62] Cheon SH, Kim KS, Kim S, Jung HS, Choi WC, Eo WK. Efficacy and safety of *Rhus verniciflua* stokes extracts in patients with previously treated advanced nonsmall cell lung cancer. Forsch Komplementmed 2011;18:77–83. 10.1159/000327306 21576976

[63] Ministry of food and drug safety in Republic of Korea. Available from: https://www.foodsafetykorea.go.kr/portal/board/boardDetail.do

[64] Youn Y, Baek HI, Jin HY, Jeong DY, Shen L, Joo JC, et al Randomized double-blind human trial to evaluate efficacy and safety of *Rhus verniciflua* Stokes (Lacca Sinica Exsiccata) and *Eucommia ulmoides* Oliver (Eucommiae Cortex) extract combination (ILF-RE) on improvement of liver function. Kor J Herbol. 2020;35(1):45–55. 10.6116/kjh.2020.35.1.45

